# Cell Scenario: A New Look at Microarrays

**DOI:** 10.1289/ehp.114-a172

**Published:** 2006-03

**Authors:** Carol Potera

The field generally known as cell-based analysis started in the 1970s with two-dimensional gel electrophoresis, which tracked levels of proteins in cells. Next came DNA microarrays that measured thousands of genes simultaneously. Today researchers are exploring the potential of a new tool, the Phenotype MicroArray™ (PM), which offers a panoramic view of cellular events. With the recent introduction of a mammalian version of the PM, the tool is poised to provide even more insight into how cells behave when affected by environmental agents.

Just like a battery of tests on a person’s blood can scan the health of vital organs, the PM can scan the physiology of cells, yielding data on hundreds of traits at once. Typical cell-based assays measure only one trait at a time (for example, cell death or DNA synthesis), but the PM can measure up to 2,000 traits—or phenotypes—under hundreds of growth conditions. The PM can be used to fingerprint cell lines used in research or bio-manufacturing to ensure stability, or monitor the effects of drugs or toxicants on cells. Researchers can also compare normal and diseased cells to see how cell functions are altered.

## Building on Success

PM developer Barry Bochner, a bioengineer, patented a simple dye method to measure cellular respiration while a graduate student in the 1970s. Ten years later, he started operations at Biolog Incorporated in Hayward, California, to commercialize the technology as a tool to identify more than 1,900 species of bacteria and fungi based on patterns of carbon metabolism. These first products, a series of five kits that each can identify about 300–600 species, have been a chief tool of microbiologists since 1988.

Microbes grow on many carbon sources, such as glucose and other sugars, amino acids, and carboxylic acids. The original microbial identification kits were based on redox reactions that produce a color change in microwells; that is, when cells utilize carbon for energy, they turn a colorless dye purple. Each well of the microbial identification kits contains a different assay—a different carbon fuel and a tetrazolium dye—dried on the bottom. When a microorganism metabolizes a carbon source, an irreversible chemical reaction occurs, and the intensity of the purple color formed in each well over time is analyzed and compared to a database for identification.

Tetrazolium dye has long been used by toxicologists to measure cell viability. Bochner’s team improved the older dye chemistry by making it water-soluble and less toxic to cells. In addition, they eliminated high background color that results from serum in culture media reacting with older tetrazolium dyes. With the improved dye, researchers can measure as few as 100 or up to 20,000 cells in one well.

Bochner’s next invention came during the genomics era, when DNA microarrays allowed scientists to measure the expression of thousands of genes simultaneously. “I had this idea that we could go beyond carbon metabolism,” says Bochner, now chairman and vice president of research and development at Biolog. So he created the PM.

The PM uses the same technology as the microbial identification kits, except that the PM measures nearly 2,000 chemical reactions related to carbon, nitrogen, phosphorus, and sulphur metabolism, as well as pH, growth range, and sensitivity to antibiotics and stress factors. The reactions reflect key cell pathways, including cell surface binding, biosynthesis of molecules, stress and repair processes, and the metabolism of carbon and nitrogen. “With two thousand phenotypes we can detect most of the important changes in cellular physiology,” says Bochner.

Few technical skills are required to run any of the Biolog kits. A researcher simply adds a cell suspension to the wells to start the reactions. The data generated are captured and interpreted by Biolog’s OmniLog® system, a combination incubator and scanner that monitors, analyzes, records, and graphs changes in each well with proprietary bioinformatic software. Data are collected in 15-minute intervals for up to 48 hours.

## PM Applications

Data from the PM and DNA microarrays complement each other and bridge the gap between molecular changes and biological outcomes. “Just because a gene is turned on or off doesn’t mean that a biological pathway gets turned on,” says Bochner. The PM gives a global view of cellular processes by detecting how gene changes alter one or many biological properties of cells.

The PM can therefore help researchers assess the effects of environmental toxicants on cells. Toxicants work by interfering with cellular respiration, damaging the pathways that cells need to live and grow, so the formation of the purple dye color is either reduced or totally prevented. Similarly, pharmaceutical companies can use the PM to monitor toxicity of new drugs. “This is a general method for studying the effects of any chemical on cell pathways,” says Bochner.

The function of novel genes can be assessed with knockout experiments to see what cell phenotypes appear, disappear, strengthen, or weaken. In knockouts of *Pseudomonas aeruginosa*, which infects the lungs of cystic fibrosis patients, investigator Ian Paulsen of The Institute for Genomic Research uncovered unusual gene transport functions. “Biolog let us pin functions to novel genes,” says Paulsen, who is mapping the physiology of this pathogen. “There is no comparable product on the market that lets you do high-throughput physiological screening.”

The PM can also highlight pathways linked to a pathogen’s virulence. At Lawrence Livermore National Laboratory, Sandra McCutchen-Maloney studies Y*ersinia pestis,* the cause of bubonic plague. *Y. pestis*, one of the most virulent bacteria known, is feared as a possible bioterrorism threat. The microbe infects rodents in North America, and fleas can transmit *Y. pestis* to humans.

When McCutchen-Maloney used the PM to test *Y. pestis* under biologically relevant conditions that occur in fleas and humans, the pathogen proved tougher than expected and less vulnerable to antibiotics. The data “uncovered new pathways involved in virulence that could be targets for future therapeutics,” says McCutchen-Maloney, who presented these findings at the 44th annual meeting of the American Society for Cell Biology in December 2004.

In another application, USDA veterinarian Jean Guard-Bouldin studied how eggs with uncracked shells become contaminated with *Salmonella*. The PM was used to “identify a number of physiological capabilities in *Salmonella* that we would not have otherwise predicted,” says Guard-Bouldin. It turned out the egg-contaminating strains had evolved metabolic capabilities that adapted the pathogen to grow in the reproductive tracts of hens that otherwise appeared healthy. The PM approach “accelerated our ability to locate the genes that were undergoing rapid evolution,” she says.

Like DNA microarrays, the PM generates massive amounts of data. “The challenge is to find ways to make sense of large data sets,” says Paulsen. It is up to bioinformatics experts to develop statistical methods to analyze data in a way that makes sense for their application.

With the new mammalian PM, introduced in September 2005 at the Society for Biomolecular Screening conference held in Geneva, Switzerland, researchers now have the ability to work with a variety of human cell lines, ranging from blood to liver cells, as well as primary rat hepatocytes, which toxicologists prefer.

The first mammalian PM contains 384 assays for energy-producing pathways shared by a range of cell types. “It was a big leap to mammalian cells,” says Bochner, who spent three years adapting the method to work in more complex mammalian cells. The goal is to expand the mammalian PM to 2,000 phenotypes as the methods are perfected.

## New Applications for the Tried-and-True

Environmental scientists are finding new applications for Biolog’s tried-and-true microbial identification kits. Ken Cullings, an evolutionary ecologist at NASA’s Ames Research Center, used one kit to evaluate the effects of defoliation on soil fungal diversity. In a mixed lodgepole pine and Engelmann spruce forest in Yellowstone National Park, Cullings’s team removed half the needles on naturally reseeded pine seedlings. A year later, soil samples showed a significant increase in the physiological diversity of soil fungi. “Fungi specific to pines jumped ship to the nondefoliated spruces when the pine was defoliated,” says Cullings.

The results, reported in the April 2005 issue of *Applied and Environmental Microbiology*, translate directly to forestry practices: after harvesting old-growth forests, replant with mixed tree species to improve future productivity. The same advice holds for replanting after forest fire devastation. “We showed in a pine–spruce ecosystem that mixed species moderate detrimental effects,” says Cullings.

Another NASA scientist, microbial ecologist Jay Garland of the Kennedy Space Center, is seeking beneficial microbes to prevent *Salmonella* contamination of alfalfa sprouts. NASA hopes to someday send alfalfa sprouts on space missions to produce oxygen and water and also serve as a food source for astronauts. But sprouts are vulnerable to *Salmonella* contamination. Since 1995, at least 21 food poisoning outbreaks due to eating contaminated sprouts have been reported in the United States, according to Garland.

To prevent infection, seeds are soaked in bleach, but the practice is neither completely effective nor palatable to health-conscious consumers. “An alternative is to manipulate the numbers of good microbes that live naturally on sprouts to stop *Salmonella* from growing,” says Garland. He uses one of the microbial identification kits to screen bacteria growing naturally on sprouts to identify mixtures that rapidly block the growth of harmful *Salmonella* and other pathogens, as described in the January 2005 issue of *Journal of Food Protection*. This biocontrol strategy could protect both Earth-bound consumers and astronauts.

James Thomas, a molecular biologist at Canada’s University of Lethbridge, performs source tracking of fecal indicator bacteria in southern Alberta, where an intense livestock industry generates enormous amounts of waste. During the summer, smaller communities often must boil water due to high levels of fecal bacteria. In collaboration with Health Canada, Thomas screens environmental water samples from the city of Lethbridge and surrounding rural communities with Biolog kits to find out how the contamination is happening, and why it especially occurs in the hot weather.

Thomas and his colleagues used a microbial identification kit to monitor 37 sites within the Oldman River Basin, where a network of irrigation canals and three reservoirs provide water to the agricultural region. The results showed that the lowest levels of the enteric bacteria *Escherichia coli* and *Enterococcus faecalis* are detected at the outflow of reservoirs. “Reservoirs remove microbial pollutants in agricultural waste-waters,” says Thomas. The construction of more in-stream reservoirs or wetlands could significantly improve the water quality of rural watersheds, they concluded in the 15 September 2005 issue of S*cience of the Total Environment*. **–Carol Potera**

## Figures and Tables

**Figure:**
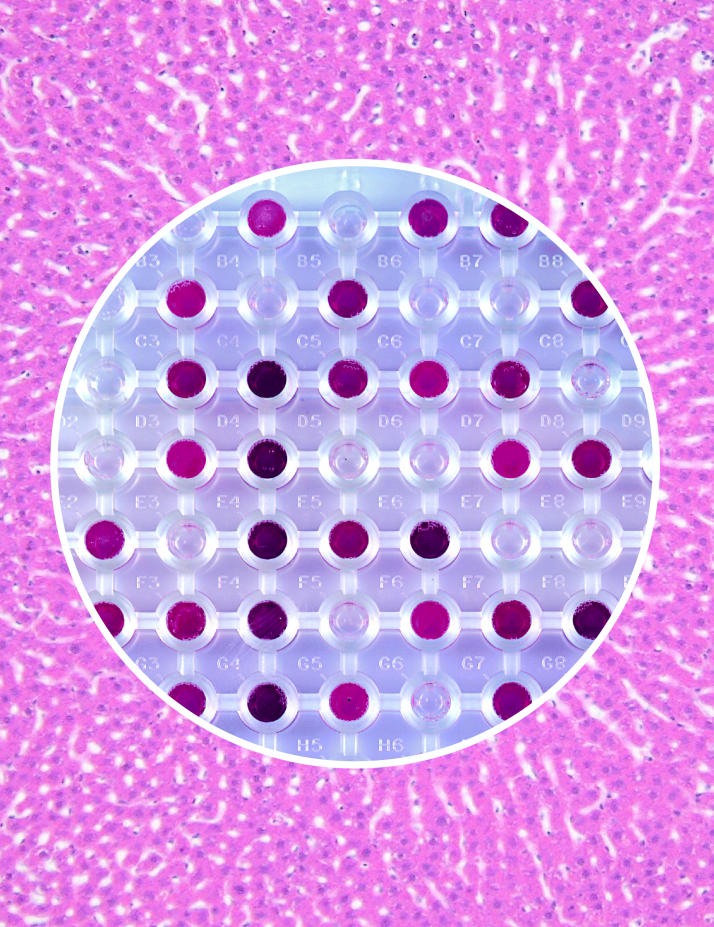


**Figure:**
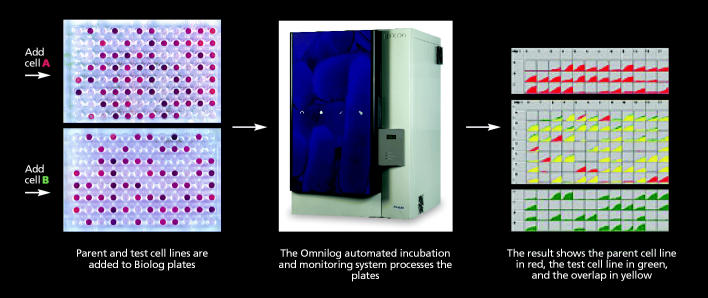
How the Phenotype MicroArray Works

## References

[b1-ehp0114-a00172] Cullings K, Raleigh C, New MH, Henson J (2005). Effects of artificial defoliation of pines on the structure and physiology of the soil fungal community of a mixed pine–spruce forest. Appl Environ Microbiol.

[b2-ehp0114-a00172] Gannon VP, Duke GD, Thomas JE, Vanleeuwen J, Byrne J, Johnson D (2005). Use of in-stream reservoirs to reduce bacterial contamination of rural watersheds. Sci Total Environ.

[b3-ehp0114-a00172] Morales CA, Porwollik S, Frye JG, Kinde H, McClelland M, Guard-Bouldin J (2005). Correlation of phenotype with the genotype of egg-contaminating *Salmonella enterica* serovar Enteritidis. Appl Environ Microbiol.

[b4-ehp0114-a00172] Zhou L, Xiang Lei X-H, Bochner BR, Wanner BL (2003). Phenotype MicroArray analysis of *Escherichia coli* K-12 mutants with deletions of all two-component systems. J Bacteriol.

